# Targeting replication stress in glioblastoma: From genotoxic treatment and inhibitors of the DNA damage response to immunotherapy

**DOI:** 10.1093/noajnl/vdag177

**Published:** 2026-07-09

**Authors:** Alexandros Pailas, Ana Catarina Vales de Almeida, Miriam Torres-Fernández, Anthony Chalmers, Michel Mittelbronn, Eric Van Dyck

**Affiliations:** DNA Repair and Chemoresistance Group, Department of Cancer Research (DoCR), Luxembourg Institute of Health (LIH), Luxembourg, Luxembourg; Faculty of Science, Technology and Communication, University of Luxembourg, Esch-sur-Alzette, Luxembourg; DNA Repair and Chemoresistance Group, Department of Cancer Research (DoCR), Luxembourg Institute of Health (LIH), Luxembourg, Luxembourg; Faculty of Science, Technology and Communication, University of Luxembourg, Esch-sur-Alzette, Luxembourg; DNA Repair and Chemoresistance Group, Department of Cancer Research (DoCR), Luxembourg Institute of Health (LIH), Luxembourg, Luxembourg; Faculty of Science, Technology and Communication, University of Luxembourg, Esch-sur-Alzette, Luxembourg; School of Cancer Sciences, University of Glasgow, Glasgow, UK; Department of Health, Medicine and Life Sciences (DHML), Faculty of Science, Technology and Medicine (FSTM), University of Luxembourg, Esch-sur-Alzette, Luxembourg; Division of Neuropathology, Department of Pathology and Neuropathology, Medical Faculty, University of Cologne, Cologne, Germany; DNA Repair and Chemoresistance Group, Department of Cancer Research (DoCR), Luxembourg Institute of Health (LIH), Luxembourg, Luxembourg

**Keywords:** chemotherapy, DNA damage response (DDR), glioblastoma (GBM), immunotherapy, replication stress response (RSR)

## Abstract

Replication stress (RS) encompasses states of impaired DNA replication progression associated with fork slowing, stalling, or collapse. RS triggers a specialized branch of the DNA damage response (DDR) called the RS response (RSR) and constitutes a major source of genomic instability, which is a key hallmark of cancer. Cancer cells face high basal levels of RS due to sustained proliferative signaling. Besides endogenous stimuli, RS can be induced by exogenous sources such as chemotherapeutic drugs. In glioblastoma (GBM), the most aggressive primary brain tumor, chronic RS leading to DDR activation is thought to drive enhanced DNA repair and treatment resistance. While chromosomal instability associated with DDR defects drives tumorigenesis, the need to preserve genomic stability to maintain fitness fosters addiction to redundant pathways promoting fork integrity. Targeting these pathways is a promising anti-cancer strategy currently undergoing clinical evaluation. In this review, we provide a molecular overview of RS and the RSR. We also explore the sources of RS in gliomas, along with the biological processes and tumor-specific features that influence the RSR in GBM cells. We then delve into the strategies developed to exploit RS, including strategies harnessing the DDR to foster antitumor immunity.

Key Points

- Replication stress—a hallmark of cancer cells—triggers a branch of the DNA damage response called the replication stress response
- Targeting replication stress in glioblastoma offers new opportunities to enhance current therapeutic approaches and exploit exposed vulnerabilities through synthetic lethality
- Harnessing the DNA damage response is emerging as a promising strategy to promote anti-cancer immunity in GBM

Targeting RS and components of the RSR in cancer cells has shown promising outcomes and is transforming the therapeutic approaches to cancer. This review provides a molecular overview of RS and the RSR, highlighting candidate therapeutic targets currently under preclinical or clinical investigations. We also explore the sources of RS in gliomas and discuss tumor-specific features and processes that foster RSR in GBM. We then describe strategies developed to exploit RS, including strategies harnessing the RSR and the DDR to foster antitumor immunity.

## Replication Stress and the Replication Stress Response

### Replication Stress

**Figure 1.**
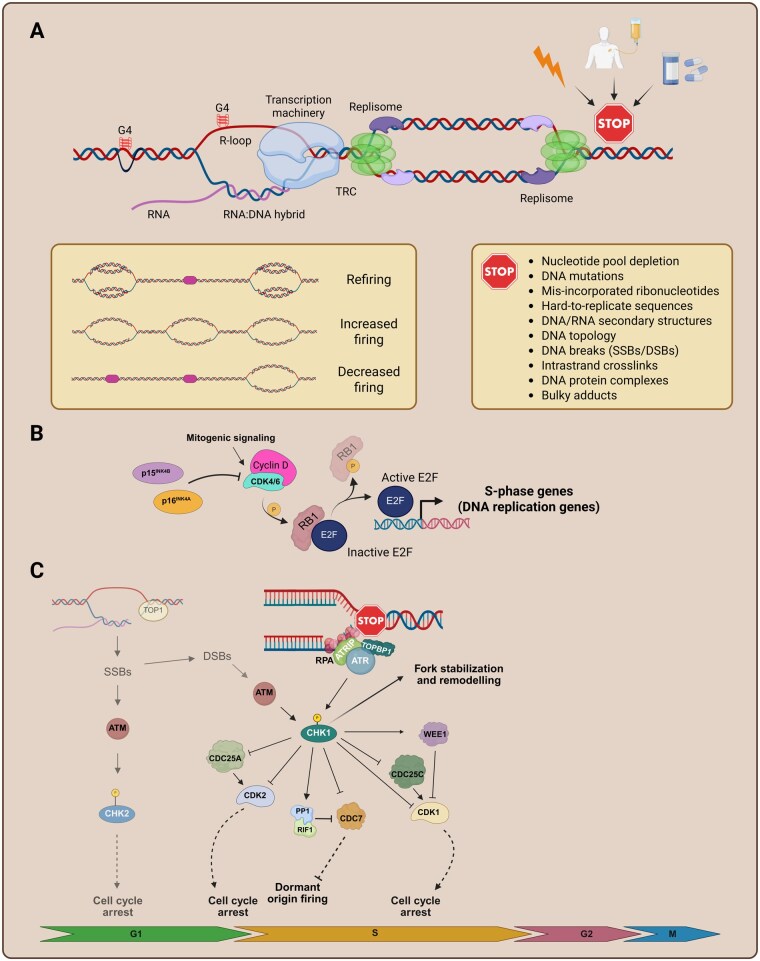
Sources of replication stress, regulation of the RB1 pathway orchestrating the G1/S transition and roles of ATR in the replication stress response. (A) Roadblocks to replicative DNA polymerases include the depletion of nucleotide pools, DNA mutations, mis-incorporated ribonucleotides and hard-to-replicate sequences such as DNA repeats and secondary DNA structures (eg, DNA hairpins and 4-stranded G-guadruplex (G4) structures formed in guanine-rich DNA sequences), as well as DNA lesions induced by chemotherapeutic DNA damaging agents (eg, DNA crosslinks induced by cisplatin, DNA-protein complexes induced by topoisomerase I (TOP1) poisons, bulky adducts induced by UV radiation, and DNA lesions induced by ionizing radiation). Other major sources of RS include TRCs, as well as R-loops. Represented is a head-on (HO) collision between the replisome and the transcription machinery (as opposed to co-directional TRCs occurring when the 2 machineries progress in the same orientation). While HO TRCs promote R-loop formation, co-transcriptional R-loops such as the one depicted here also exacerbate the impact of TRCs on RS. In addition, R-loops are vulnerable to nucleases such as XPF-ERCC1 and XPG, which can lead to SSBs and DSBs. Also shown are examples of activated oncogene-induced deregulation of fork origin firing. (B) Depiction of the RB1 regulation pathway governing the G1/S transition. See text for details. (C) Under RS, the accumulation of RPA-coated ssDNA leads to the ATRIP-mediated recruitment of ATR and its activation. ATR phosphorylates and activates the CHK1 signaling kinase, which mediates the inhibition of CDC25 phosphatases and parallel activation of the WEE1 kinase, leading to the loss of CDK1/2 activity and halting cell cycle progression at the intra-S and G2/M checkpoints. The ATR-CHK1 axis also inhibits excessive origin firing through the direct, inhibitory phosphorylation of the S-phase kinase CDC7, as well as by promoting formation of the PP1-RIF1 complex, which inhibits CDC7. ATR-Chk1 activates a sequence of events leading to fork stabilization/remodeling (detailed in Figure 2). Illustrated on the left side of the panel is the transcription-associated DNA damage (SSB) elicited by the TOP1 poison CPT, leading to ATM-mediated signaling in G1. When carried into S-phase, SSBs lead to DSBs.

RS refers to states of impaired DNA replication progression associated with fork slowing, stalling, or collapse (reviewed in Refs^1-5^). RS is a hallmark of cancer cells where it is induced by sustained proliferative signaling, as well as high transcription rates associated with genetic and structural alterations affecting oncogenes and tumor suppressor genes.^3^^,^^6-9^ The deregulation of replication fork progression by activated oncogenes can manifest as unscheduled firing of replication origins (ie, increased or decreased firing or refiring of the same origin), deregulated nucleotide and reactive oxygen species (ROS) metabolism, or increased transcription activity.^1^ The deregulation of these processes impacts fork speed, fork collisions, and the depletion of deoxynucleotide pools and crucial replication factors like RPA. Additionally, it can trigger the accumulation of R-loops (three-stranded structures that are formed when a newly transcribed RNA molecule hybridizes with the DNA template strand, thus extruding the non-template strand)^10^ and transcription-replication conflicts (TRCs)^1^ (Figure 1A).

Among the prominent sources of RS in cancer cells is the deregulation of the G1/S transition, a process under tight regulation from the master regulator of the cell cycle, RB1 (RB Transcriptional Corepressor 1) (Figure 1B). Under physiological conditions, p16^INK4a^ (encoded by *CDKN2A*) and p15^INK4b^ (encoded by *CDKN2B*) maintain RB1 in a hypo-phosphorylated state by inhibiting cyclinD-CDK4/6 complexes. RB1 in this state binds to members of the E2F transcription factor family, preventing unscheduled transcription of genes required for S-phase, including DNA synthesis genes, and preventing entry into S-phase. Under normal conditions, mitogenic signaling in early G1 is sufficient to surpass the inhibitory effect of CDKN2A/B, leading to the inactivation of RB1 via hyperphosphorylation and degradation, as well as the release of E2F to activate the S-phase transcription programs and signal the G1/S transition.^11^^,^^12^ Deregulation of the RB1 pathway through loss of p16^INK4a^/p15^INK4b^, constitutive activation of CDK4/6, or RB1 inactivation results in the unchecked activity of E2F transcription factors, primarily E2F1, leading to the premature expression of replication fork assembly and firing factors and S-phase entry. This premature S-phase entry induces severe RS through deregulated origin firing and subsequent dNTP depletion, as well as increased TRCs.^1^^,^^12-17^

Notably, although the spatio-temporal dynamics of replication and transcription partially contribute to attenuate TRCs in mammalian cells, long genes that require more than 1 cell cycle to complete transcription are more prone to TRCs.^18^ These include long genes associated with common fragile sites (CFSs)—that is difficult-to-replicate loci prone to chromosomal breakage and associated with chromosomal rearrangements and viral integration in cancer cells.^19^

Other endogenous and exogenous sources of RS have been identified, such as trapped protein-DNA complexes induced by chemotherapeutic drugs, DNA breaks and other lesions induced by genotoxic agents, including ionizing radiation (IR),^20^ and DNA/RNA secondary structures including 4-stranded DNA structures called G-quadruplexes (G4) that arise in certain guanine-rich sequences^21^ (Figure 1A).

### Replication Stress at Telomeres

Telomeres, the physical ends of our chromosomes, display several structural features that render them highly susceptible to RS (reviewed in Refs^22^^,^^23^). Telomeres consist of long, tandem arrays of TTAGGG repeats,^24^ embedded in an unusual heterochromatin environment,^25^ that are transcribed into long noncoding (lnc) telomeric RNAs called TERRA (Telomeric Repeat-containing RNA).^26^ TERRA lncRNAs can generate telomeric R-loops. Besides, the guanine-rich nature of telomeres makes them prone to oxidative damage,^27^ as well as promoting the formation of G-quadruplexes,^28-30^ that hamper replication and transcription. Finally, telomeres need protection from DNA degradation and from being recognized as DNA ends by the DNA double-strand break (DSB) repair machinery, whose action would elicit chromosome end-to-end fusion and genomic instability.^31^ Telomere end-protection involves the TERRA lncRNAs, as well as a protein complex called shelterin,^31^ that facilitates the formation of a t-loop—a lariat structure that results from a recombination mechanism whereby the telomeric single strand (ss) overhang at the extremity of telomeres loops back to invade the duplex telomeric DNA. During this process, the telomeric ss overhang itself displaces 1 strand of the duplex DNA, generating a displacement (D)-loop.^32^ The t-loop and D-loop structures must be unwound to allow telomeric DNA replication and prevent telomere erosion.^33^^,^^34^

To achieve replicative immortality, most cancers maintain telomere length by telomerase re-activation mechanisms, including through mutations in the *TERT* gene promoter, but ∼10%-15% of cancers use a telomerase-independent, DNA recombination-mediated alternative mechanism called ALT (alternative lengthening of telomeres).^35^ TERRA and telomeric R-loops are essential to promote ALT,^36^^,^^37^ but their levels must be finely tuned to preserve telomere integrity.^38^ Telomeric ALT is a feature of pediatric high-grade gliomas (pHGGs) presenting with H3.3 mutations and loss of the histone H3.3 chaperone ATRX/DAXX,^35^ as well as of IDH-mutant astrocytomas presenting with ATRX loss (see below).^39^

### Replication Stress Response

To preserve replication fork and genome integrity, cells have evolved a dedicated branch of the DDR^40^^,^^41^ known as RSR, which implements cell cycle checkpoints, including an intra-S phase checkpoint, as well as the inhibition of replication origin firing and complex fork repair/protection machineries, to complete DNA synthesis (reviewed in Refs^3^^,^^42^). ATR is the apical kinase in the RSR.^3^ Along with the other DDR kinases ATM and DNA-PK, ATR belongs to the family of phosphoinositide 3-kinase (PI3K)-related kinases (PIKKs) that coordinate the sensing and signaling of DNA lesions with cell cycle arrest and DNA repair.^43^ ATR is activated by single-stranded DNA (ssDNA) tracts that accumulate due to helicase/polymerase uncoupling at stalled forks or to nucleolytic processing of DSBs—for example during DNA end resection, a process that provides ssDNA substrates for homologous recombination (HR)-mediated DSB repair.^43^^,^^44^ The ssDNA is rapidly coated by the heterotrimeric ssDNA binding protein RPA and this nucleoprotein complex recruits ATR via its partner ATRIP, allowing ATR activation by TOPB1 or ETAA1.^43^^,^^45^ In contrast, ATM is recruited and activated by the MRN (MRE11-RAD50-NBS1) complex at DSBs that form upon replication fork collapse, to promote their repair by HR.^43^ ATR and ATM usually signal via checkpoint kinase 1 (CHK1) and CHK2, respectively, but ATM can interact with ATR signaling, for example to activate ATR-CHK1 in response to DSBs.^46^ DNA-PK is involved in several aspects of the DDR, playing a major role in mediating DSB repair by non-homologous end-joining (NHEJ), as well as mediating RPA32 phosphorylation at Ser4/8—which controls DNA resection. Additionally, it controls fork dynamics and the formation of reversed replication forks and serves as a backup for ATR during the RSR.^47-50^

**Figure 2.**
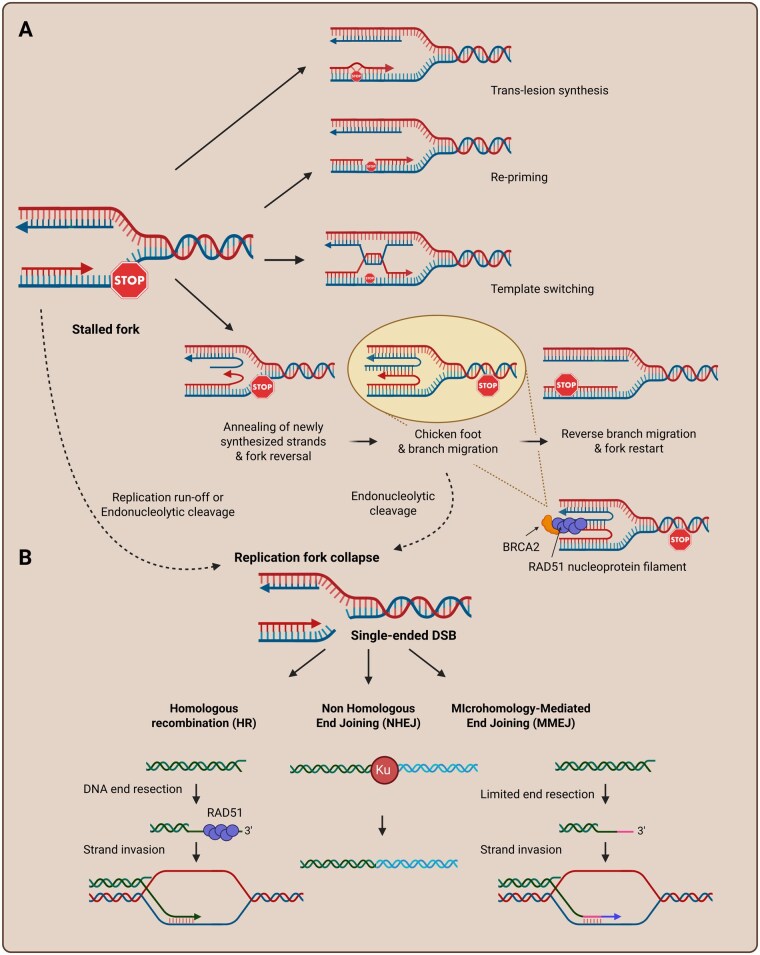
Replication fork damage and restoration. (A) Shown is a stalled fork due to an obstacle on the DNA template and DNA damage tolerance pathways. TLS uses specialized, error-prone DNA polymerases capable of inserting bases opposite a damaged template. Re-priming mechanisms mediated by PrimPol (endowed with primase and polymerase activity) allow de novo DNA synthesis past the lesion. Template switching involves strand invasion into the newly replicated strand, which is used as a template. Fork reversal involves the formation of a 4-way junction called the chicken foot structure by the re-annealing of the parental strands and the annealing of the newly synthesized strands. This reaction is mediated by DNA translocases and the RAD51 recombinase, promoting, respectively, the annealing of RPA-coated ssDNA and strand-exchange activity resulting in a newly formed duplex. Other key DDR factors promoting chicken foot formation are the RAD51 recombinase, the DNA translocases SMARCL1, ZRANB3, and HLTF, DNA helicases such as BLM and WRN, RPA, Fanconi Anemia factors, and PARP1. In a novel reaction where BRCA2 acts as a mediator, RAD51 assembles nucleoprotein filaments on the regressed arm of the chicken foot, thus protecting it from nucleolytic resection. This structure can serve as a substrate for branch migration by DNA translocases. Note that in the reverse fork, the replicative DNA helicase is pushed away from the lesion but still bound to ssDNA, thus poised for replication restart upon reverse branch migration. Alternatively, cleavage of the chicken foot by structure-specific endonucleases results in DSBs. (B) Several DSB repair pathways can operate on seDSBs. HR is initiated by RAD51, which assembles nucleoprotein filaments on 3′-ended ssDNA tails generated by resection of the DSB and mediates strand invasion into a homologous duplex. NHEJ entails binding and juxtaposition of distant DSB ends (represented by different colors) by the DNA end-binding factor Ku, followed by ligation of the ends. POLQ-mediated MMEJ (shown here at a broken leading strand) entails DNA unwinding, the search for microhomologies (MH, in blue) and their annealing, followed by DNA synthesis.

The salient features of the ATR pathway are summarized in Figure 1C. A prominent role for ATR is to arrest the cell cycle and restrict the firing of all dormant replication origins (ie, origins that have been licensed for replication but not activated) to prevent ssDNA accumulation and avoid replication catastrophe due to RPA exhaustion (Figure 1C).^45^^,^^51^ Notably, certain RS-inducing drugs such as camptothecin (CPT) not only impede replication but also induce transcription-associated DNA damage that may itself elicit DSBs upon replication, activating both ATR and ATM pathways (Figure 1C).^52^^,^^53^ Another role for ATR is to control the remodeling of stalled forks, for example, in front of a DNA lesion or due to unbalanced deoxynucleotide pools, to foster either fork reversal/restart,^54^ or DNA damage tolerance mechanisms such as translesion DNA synthesis (TLS), replication fork repriming and template switching, that allow obstacles to be overcome with a minimal impact on fork elongation (Figure 2A).^55^ ATR also plays a crucial role in the signaling and handling of collapsed replication forks and the associated DSBs (Figure 2B). Although DSBs can be generated by structure-specific endonucleases (eg, MUS81-EME1/EME2) as part of the normal process leading to replication fork restart,^56^ they can also result from aberrant protection or processing of stalled/reversed forks leading to fork collapse. DSBs can also form through replication “running off” upon encounter with SSBs (such as those resulting from temozolomide-induced O6-meG lesions left unrepaired by MGMT (see below)), or with protein-DNA complexes (such as those trapped by the poly(ADP-ribose) polymerase 1 (PARP1) inhibitor olaparib or the topoisomerase I (TOP1) poison CPT) (Figure 2B).^57-59^

Replication fork collapse leads to a special type of DSB called single-ended DSB (seDSB).^49^ Recombinational repair provides the major route for seDSB repair during the S and G2 phases of the cell cycle,^60^^,^^61^ through a RAD51-dependent DNA replication pathway called break-induced replication (BIR) that acts on 3′ ssDNA tails generated by DNA end resection (Figure 2B).^61-63^ DSBs at collapsed replication forks can also be processed by non-homologous end-joining (NHEJ),^49^^,^^64^^,^^65^ a toxic mechanism that involves the bridging and sealing of distant DNA ends (Figure 2B), resulting in chromosomal aberrations and genetic instability.^66^ However, fully active HR outcompetes NHEJ for seDSB repair in S/G2, thus preventing genome instability.^67^ seDSBs can also be processed by microhomology-mediated end joining (MMEJ), a mutagenic pathway mediated by the DNA helicase-polymerase theta (POLQ), that anneals short microhomologies exposed by 3′ ssDNA overhangs and extends the annealed microhomologies using the opposite strand as template (Figure 2B).^68^^,^^69^

Finally, as is the case for DNA repair, the RSR entails massive chromatin rearrangements and changes in histone dynamics and modifications.^70^^,^^71^

### Processing of RS-Associated Lesions during Mitosis: Preventing Chromosomal Instability

RS-associated DNA lesions such as under-replicated DNA and unresolved DNA structures can evade cell cycle checkpoint surveillance or be tolerated (due, eg, to their low levels) during interphase and persist until cells enter mitosis, with potentially catastrophic consequences.^72^ Alongside mitotic dysfunction of the machineries that ensure sister chromatid cohesion, the assembly and dynamics of the mitotic spindle apparatus and maintenance of the spindle assembly checkpoint,^73^ RSR dysfunction and/or failure to address unresolved RS lesions during mitosis represent major sources of chromosomal instability (CIN), including defective chromosome segregation, micronuclei, and aneuploidy. For instance, under-replicated DNA regions, including common fragile sites (CFSs), have the potential to generate ultra-fine DNA bridges (UFBs) in anaphase cells^74^ or micronuclei (MN)—membrane-bound structures separated from the main nucleus that encapsulate DNA and arise during mitosis from lagging chromosomes or chromosomal fragments, following missegregation or the accumulation of unrepaired or incompletely replicated DNA—in the daughter cells.^75^

The processing of DNA lesions during mitosis is achieved by several “salvage” mechanisms: (i) mitotic DNA synthesis (MiDAS), a BIR mechanism that requires the RAD52 recombinase, as well as the structure-specific endonuclease MUS81-EME1 and POLD3 to complete duplication of under-replicated DNA regions,^76^ (ii) mechanisms relying on the BTR complex (BLM DNA helicase, Topoisomerase IIIα, RMI1-RMI2), the SMX tri-nuclease complex (composed of SLX4-SLX1, MUS81-EME1 and XPF-ERCC1) and the endonuclease GEN1, to disengage or resolve covalently-linked 4-way DNA junctions called Holliday junctions that are formed during DNA recombination,^77^^,^^78^ and (iii) topoisomerase IIα to decatenate braided sister chromatids.^79^

## Sources of Replication Stress in Gliomas

**Figure 3.**
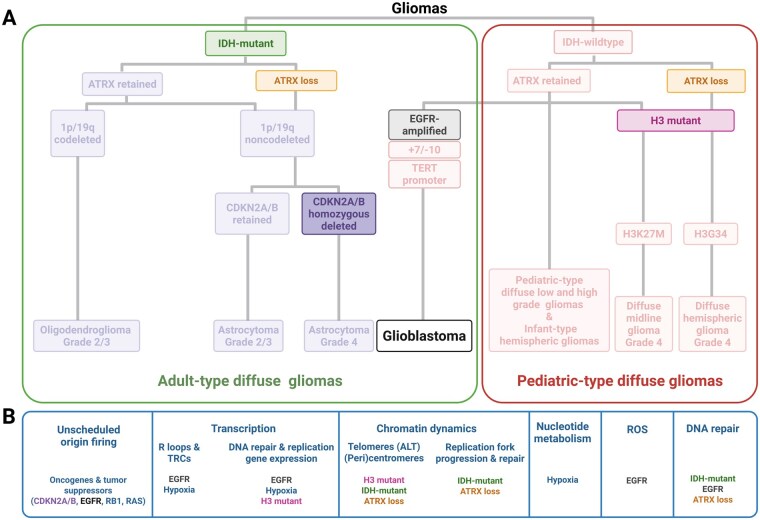
Sources of replication stress driven by key glioma molecular alterations. (A) 2021 WHO classification of diffuse gliomas highlighting key alterations associated with RS. (B) How these alterations affect the various sources of RS to alter crucial cellular programs is presented. Although unrelated to the glioma classification, the impact of hypoxia on RS is also presented. See text for details.

Advances in our understanding of the molecular alterations underlying diffuse gliomas have led to a novel WHO classification of diffuse gliomas that refines established approaches to tumor diagnosis by integrating molecular markers, including isocitrate dehydrogenase (*IDH*) 1 and 2 mutations, 1p/19q co-deletion, *EGFR* amplification, and mutations affecting *ATRX*, as well as the *TERT* promoter and genes encoding histone H3 and its variant H3.3.^80-82^ How some of these alterations affect RS is starting to emerge (Figure 3).

### Replication Stress in Mutant IDH Gliomas

The *IDH* mutations found in astrocytoma, IDH-mutant, grade 2 or 3, mostly represented by heterozygous *IDH1* (R132H), confer a neomorphic enzymatic activity. Specifically, whereas wild-type (WT) IDH catalyzes the conversion of isocitrate into α-ketoglutarate (α-KG), mutant IDH converts α-KG into the oncometabolite 2-hydroxyglutarate (2HG)—an inhibitor of multiple αKG-dependent dioxygenases, including DNA/RNA demethylases, histone demethylases and proline/lysine hydroxylases.^83^^,^^84^ In addition to metabolic alterations,^85-87^ the accumulation of 2HG impacts the removal of crucial epigenetic marks, leading to alterations in a variety of cellular programs affecting cellular metabolism, cancer biology and oncogenesis.^83^^,^^87^^,^^88^

Mutant IDH alters the DDR, including through inhibition of the DNA repair enzymes ALKBH2/3 involved in the removal of alkylating lesions such as those induced by temozolomide,^89^ as well as suppressing homologous recombination (HR) by impacting the methylation of histone H3 lysine 9 required for the recruitment of HR factors at DSBs (Ref^90^ and refs therein). Moreover, mutant IDH confers sensitivity to RS-inducing drugs.^91^ Recently, genetic screens targeting αKG-dependent enzymes identified KDM6 histone demethylases (which remove H3K27 methylation marks) as crucial determinants of the RS response inhibited in mutant IDH cells,^92^ in line with the notion that fine-tuning of H3K27 methylation is required to promote replication fork restart and maintain genomic stability.^93^ It is noteworthy that the global loss of H3K27me3 in H3K27M and H3.3K27M mutant glioma also confers RS,^94^^,^^95^ which is associated with hypertranscription in H3K27M glioma,^94^ further highlighting a critical role for proper H3K27 methylation in the prevention of RS.

### RS in Mutant ATRX Gliomas

Loss-of-function mutations in *ATRX* (α-thalassemia/mental retardation X-linked) occur in 75% of adult IDH mutant grade 2/3 astrocytomas,^96^^,^^97^ and in around 30% of pediatric gliomas characterized by histone H3.3 mutations.^98-100^

ATRX, in partnership with DAXX, forms a histone chaperone that deposits the H3.3 variant at specific regions of our chromosomes, including (peri)centromeres, telomeres, and endogenous retroviral elements (ERVs) that are characterized by the presence of repetitive DNA sequences and constitutive heterochromatin. Importantly, H3.3 provides the heterochromatic H3K9me3 mark in these regions.^100-102^ In the ATRX/DAXX complex, DAXX provides the H3.3-chaperone activity while ATRX acts as a chromatin remodeler. By orchestrating transcriptional regulation, chromatin remodeling, heterochromatin maintenance and DNA repair, the ATRX/DAXX complex serves as a key guardian of genomic stability.^96^^,^^99^^,^^103^ In addition to forming an H3.3 chaperone complex, ATRX and DAXX have each evolved mutually independent functions in the regulation of gene expression, chromatin dynamics and DNA repair (Ref^104^ and references therein).

ATRX plays crucial roles in preventing RS in gliomas. By promoting a heterochromatin environment through deposition of H3.3, ATRX/DAXX limits G-quadruplex-induced RS.^96^ In addition, ATRX, together with TOP2B, directly mediates the resolution of G-quadruplexes during DNA replication.^99^^,^^105^ Besides, inactivating ATRX mutations are strongly associated with ALT,^106^ implicating ATRX as a suppressor of the ALT mechanism.^107^^,^^108^ However, ATRX defects alone are not sufficient to activate ALT; instead, H3.3G34R and IDH1/2 mutations have been identified as cooperating factors to drive ALT in ATRX-mutated gliomas.^109^ Noteworthy, both mutations inhibit the activity of the histone lysine demethylase KDM4B (which removes the heterochromatin mark H3K9me3) at telomeres, while knockout or inhibition of KDM4B led to increased RS and DNA damage at telomeres.^109^

### Homozygous Deletion of CDKN2A/B

As detailed above, the *CDKN2A* and *CDKN2B* genes encode crucial regulators of RB1 (RB Transcriptional Corepressor 1), a master regulator of the cell cycle (Figure 1B). In diffuse astrocytic tumors, the homozygous deletion of *CDKN2A/B* defines grade 4 astrocytomas.^110^ Additionally, such deletions, together with *CDK4* amplification and *RB1* homozygous deletion, are the most common alterations affecting the RB1 pathway to drive unchecked cell proliferation in GBM.^111^

## Replication Stress in Glioblastoma

Glioblastoma (GBM), IDH-WT, WHO grade 4, is the most prevalent and aggressive primary brain tumor, with an overall median survival of ∼15 months upon diagnosis.^112^ The current standard of care for GBM includes maximal surgical resection, followed by radiotherapy plus concomitant and maintenance chemotherapy with the DNA alkylating agent temozolomide (TMZ).^113^ Unfortunately, patients inevitably experience tumor progression and relapse due to (i) the infiltrative nature of GBM, which prevents complete surgical resection, (ii) the multi-level, heterogeneous nature of GBM and the phenotypic plasticity of GBM cells, (iii) the occurrence of intrinsic and acquired treatment resistance mechanisms, and (iv) the crucial lack of therapeutic options for recurrent GBM when the first-line treatment has failed.^114-117^ Compounding these challenges, GBM is defined as an “immunologically cold” tumor that resists treatment with current immunotherapies (this topic is developed in the last section of this review).

### Impact of EGFR Alterations on Replication Stress in Glioblastoma

Key GBM driver mutations and copy number alterations shape GBM biology and treatment resistance.^118-120^ The molecular landscape of glioblastoma (GBM), IDH-WT, includes aberrant activation of numerous signaling pathways associated with receptor tyrosine kinases (RTKs) and, in particular, the epidermal growth factor receptor (EGFR).^121^^,^^122^ EGFR signal amplification, which results in the activation of signaling pathways such as MAPK, PI3K/AKT, and mTOR to promote cancer cell proliferation,^120^ is expressed in around half of the GBM patients.^123^ This aberrant activation can occur through ligand-dependent or ligand-independent mechanisms that include lack of receptor degradation, crosstalk with other receptors, gene amplification leading to increased protein levels, and increased ligand production.^122^

Half of the patients with increased EGFR activity express a variant form of EGFR called EGFRvIII, characterized by an in-frame deletion of *EGFR* exons 2-7 (encoding part of the extracellular domain), which results in constitutive activation and lack of internalization.^123^ EGFRvIII promotes DNA repair in GBM cells, including the hyperactivation of DNA-PK via PI3K-AKT1 pathway, leading to IR resistance.^123^^,^^124^ However, by increasing the expression of key DNA mismatch repair (MMR) proteins, EGFRvIII also confers increased TMZ sensitivity and prolonged survival in GBM patients with O6-methylguanine-DNA methyltransferase (MGMT) promoter-methylated tumors (detailed in the section below).^125^ EGFRvIII expression increases the levels of ROS,^126^ and has been shown to drive the oncogenic phenotype by increasing transcription activity, resulting in the accumulation of R-loops and RS.^127^ Notably, although hyperactive or mutated EGFR elicits RS, EGFR itself appears to mitigate RS to prevent catastrophic DNA replication failure. Thus, EGFR substrates include the molecular chaperone HSP70, whose phosphorylation by EGFR facilitates the loading of the processivity factor proliferating cell nuclear antigen (PCNA) on chromatin, thus promoting the recruitment of DNA replication proteins to prevent excessive DNA replication stress.^128^ Finally, EGFR activation has also been shown to affect several DNA repair pathways,^122-124^^,^^129-131^ through a variety of mechanisms including the physical interaction of mutant forms of EGFR with DNA repair proteins,^124^^,^^130^ or the transcriptional upregulation of DNA repair genes through EGFR-mediated activation of the MEK/ERK signaling pathway.^131^

### Cellular Plasticity, DDR/RSR Activation, and Treatment Resistance in GBM

The intra-tumoral heterogeneity of GBM samples is illustrated by the presence of multilevel plasticity potentially driving chemoradiation resistance.^116^^,^^132-135^ The increased ionizing radiation (IR) resistance displayed by subpopulations of GBM (reviewed in Refs^116^^,^^136-138^) has been linked to the preferential activation of DNA damage checkpoints and increased DNA repair.^139^^,^^140^ These studies also highlighted the potential of CHK1/2 inhibition or ATM inhibition to abrogate IR resistance.^139^^,^^140^ Aberrant constitutive activation of the DDR fueled by RS has been documented in GBM clinical specimens and cell lines.^141^ Moreover, chronic RS leading to constitutive activation of the DDR has been proposed to drive the enhanced DNA repair and radioresistance observed in GBM cell subpopulations in which targeting RS by combined inhibition of ATR and PARP elicited cytotoxicity and abrogation of IR resistance.^140-142^ These resistant cell subpopulations exhibited DSBs associated with TRCs and R-loops, thus suggesting TRCs as a source of RS.^142^ The addiction of GBM cells to RS-protecting pathways has been illustrated by the observation that *BRCA1* displayed a tumor-promoting role in GBM by ensuring the transcriptional regulation of *RRM2* (encoding the regulatory (β) subunit of ribonucleotide reductase involved in regulating deoxynucleotide pools) and protecting GBM cells from endogenous RS.^143^

### MGMT Prevents seDSB Formation and RS

The DNA repair protein MGMT defines a direct repair mechanism that reverses the most cytotoxic lesion induced by TMZ, O6-methylguanine (O6-meG) (reviewed in Ref^117^). *MGMT* gene promoter methylation represents a crucial clinical biomarker in GBM.^144^ O6-meG lesions left unrepaired by MGMT elicit replication fork collapse and seDSBs in a mismatch repair (MMR) pathway-dependent manner.^145^^,^^146^ The other alkylating lesions induced by TMZ, which are repaired by BER^117^, can also lead to seDSBs when replication forks collide with BER-generated SSB intermediates.^147^ TMZ therefore induces RS not only in the 40%-50% of GBM patients where epigenetic silencing of *MGMT* is observed,^144^ but also in MGMT-positive GBM cells undergoing MGMT exhaustion or cells where replication interferes with BER.

Highlighting the impact of TMZ on RS, a recent study investigating GBM plasticity induced by TMZ identified RRM2 as a targetable factor driving GBM resistance to TMZ.^148^ Likewise, RNAi screens of GBM cells for TMZ sensitizers have uncovered HR factors involved in seDSB repair,^65^^,^^149^ suggesting that targeting of these factors may foster the efficacy of TMZ. Noteworthy, acquired resistance to TMZ was associated with increased HR and MMEJ activity in GBM cell lines and patient-derived xenograft models.^150^

### Impact of the Perivascular and Hypoxic Regions on DDR/RSR

GBMs are characterized by angiogenesis and pseudopalisading necrosis, with perivascular hypoxic regions.^151^ The interactions between perivascular tumor cells and the microenvironment foster tumor cell migration and GBM invasion.^152^^,^^153^ An epithelial-to-mesenchymal (EMT)-like program driven by the tumor cells and affecting brain microvascular pericytes—vessel-associated mural cells required for the formation and integrity of the blood-brain-barrier ^154^^,^^155^ has been proposed to describe the phenotypic changes that distinguish normal pericytes covering vessels outside of the tumor core from those covering GBM-associated vessels in the tumor core.^156-158^ Noteworthy, pericytes have been shown to potentiate the DDR in perivascular GBM cells via CCL5-CCR5 signaling and the activation of an AKT-DNA-PKcs pathway, leading to TMZ resistance.^159^

While hypoxia inhibits HR,^160^ it also induces RS and ATR/ATM signaling, but in a manner that does not lead to detectable DNA breaks even after prolonged exposure.^161^ The RSR signaling seen under hypoxia appears to protect replication forks, with short periods of hypoxia followed by reoxygenation allowing replication fork restart, contrary to prolonged periods that may cause the disassembly of the replisome.^162^ Hypoxia induces the depletion of deoxynucleotide pools due to the fact that oxygen is required for ribonucleotide reductase to oxidize the di-iron center present in its β subunit (RRM2).^162^ Recently, hypoxia was also shown to induce reactive oxygen species (ROS)-associated R-loops that accumulated in the absence of detectable DNA damage, leading to transcriptional stress.^163^

### The Subventricular Zone Fosters Protection from DNA Damage and RS

GBM cells infiltrating the parenchyma often escape surgical resection and drive tumor recurrence. The subventricular zone (SVZ) of the lateral ventricles—the largest neurogenic region in the adult brain—has received particular attention over the years as a hideout for glioma cells that contributes to treatment resistance and tumor relapse.^164^^,^^165^ Two routes promote glioma cell invasion of the SVZ. The first one involves a CXCL12-CXCR4-Aurora A axis, with CXCR4-positive GBM cells migrating through the corpus callosum in response to a CXCL12 gradient to colonize the SVZ.^166-169^ In the second one, uncovered in pediatric diffuse midline glioma, H3K27M mutant, SVZ-resident neural precursor cells (NPCs) secrete a chemoattractant complex composed of pleiotropin, HSP90 and SPARC/SPARCL1, which activates Rho/ROCK signaling in glioma cells to promote invasion.^170^

Multiple factors are thought to drive the IR resistance of glioma cells located in the SVZ. Single-cell RNA-sequencing revealed a ZEB1-centered signature in the tumor cells of the SVZ and underlined the presence of tumor supportive microglia in the SVZ microenvironment.^171^ Besides, SVZ-released CXCL12 upregulated the mesenchymal phenotype and mediated their IR resistance in vitro.^167^ Finally, a direct role for SVZ-secreted CXCL12 in fostering IR protection through activation of the MAP kinase phosphatase 1 (MKP1) was uncovered, which promoted cell survival, RAD51 stabilization and DNA repair.^172^

## Targeting RSR and DDR in Glioblastoma

### Replication Stress as a Targetable Hallmark of Cancer

While RS-induced genomic instability stemming from DDR defects drives tumorigenesis, cancer cells must maintain sufficient genomic stability to preserve fitness. Consequently, they become reliant on redundant pathways for replication protection and repair.^173^ Targeting these pathways is a promising approach in cancer therapy, which is beginning to be explored in the clinic.^4^^,^^173-175^

### Synthetic Lethality and Inhibitor-Based Strategies

**Figure 4.**
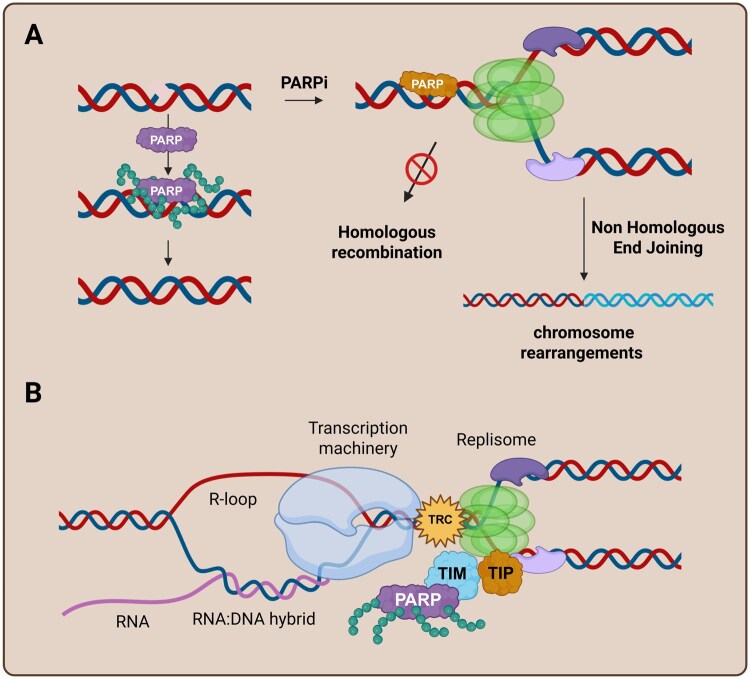
Mechanisms of action of PARP inhibitors. (A) PARP1 senses SSBs in a reaction that involves its poly (ADP-ribosylation) (represented by jade beads), a modification that acts as a platform for the recruitment of DNA repair factors such as XRCC1. PARPi trap PARP1 on the damaged DNA, resulting in seDSBs upon encounter by the replication fork. In HR-defective backgrounds, NHEJ processing of seDSB elicits chromosome rearrangements and cell death. (B) New evidence indicates a crucial role for PARP, together with TIMELESS (TIM) and TIPIN (TIP) in sensing and signaling TRCs, thus averting DSBs. Inhibition or depletion of PARP1 results in DSB that become lethal in HR-deficient cancer cells.

Among the approaches being developed to target RS are synthetic lethality strategies that take advantage of the specific DDR/RSR vulnerabilities exposed by cancer cells, by way of small molecule inhibitors.^176^^,^^177^ Underlying the concept of synthetic lethality is the notion that the individual inactivation of 2 genes (through deletion, depletion, or inhibition) is compatible with cell viability, whereas the combined inactivation of these 2 genes elicits cancer cell death. Complementing this notion is the concept of conditional synthetic lethality whereby synthetic lethal interactions are observed only under certain conditions, for example, in a specific genetic background or following exposure to DNA-damaging agents.^178^ The clinical translation of RS targeting is best illustrated by the synthetic lethality of PARP inhibitors (PARPi) in HR-deficient cancer cells. Given the role of PARP1 in SSB repair, the mechanistic basis underlying PARPi was long thought to be their action in trapping PARP1 at SSBs and inducing seDSBs upon encounter by replication forks, which would become lethal when processed by NHEJ in HR-deficient cancers (reviewed in Refs^179^^,^^180^) (Figure 4A). However, PARP1 recently appeared to operate with TIMELESS and TIPIN to protect the replisome in early S-phase from TRCs, and a novel model was proposed whereby the synthetic lethality mediated by PARPi in HR-deficient contexts results from failure to avert TRCs-induced DSBs (Figure 4B).^181^ Small-molecule PARP inhibitors are now available as first-line or second-line therapy in breast, prostate, ovarian and pancreatic cancers,^182-184^ and are in clinical trials in GBM (see below).

DDR inhibitors under investigation or clinical trials in cancer, including inhibitors of chromatin modifying factors participating in the DDR, as well as inhibitors of mitotic DNA repair mechanisms, form a rapidly expanding list.^185-188^ DDRi are being considered not only as a monotherapy, but also in combination therapy, for instance with RS-inducing chemotherapeutic drugs or immune checkpoint blockade (see below). Table 1 provides a list of the DDRi investigated in GBM, including DDRi that target checkpoint axes (eg, ATR-CHK1-WEE1), but also DNA replication and fork protection/repair factors (eg, RAD51, SMARCAL1, PARP1), as well as components of DNA repair machineries (eg, TDP1).

**Table 1.** List of RSR/DDR factors (and related inhibitors or poisons) currently under investigation or in clinical trials in GBM

General targets	Specific targets	Inhibitors/drugs	References	Clinical trials in GBM
Checkpoint axes	ATR (ATR checkpoint kinase)	Gartisertib	^189^	-
		Berzosertib	^190^	-
		AZD6738, VE-822	^191^	-
	ATM (ATM serine/threonine kinase)	AZD1390	^150^ ^,^ ^192^ ^(a,b)^	NCT05182905, NCT03423628, NCT03970447
		KU-55933	^140^ ^,^ ^193^	-
		WSD0628	^194^ ^,^ ^195^	NCT05917145
	PRKDC (protein kinase, DNA-activated, catalytic subunit)	KU57788	^196^	-
		NU7441	^197^	-
		Peposertib/M3814	^198^	NCT04555577
		CC-115	^199^	NCT02977780
	CHEK1 (checkpoint kinase 1)	UCN-01, AZD7762	^200^	-
		SAR-020106	^201^	-
		AZD7762, LY2603618 (Rabusertib)	^202^ ^,^ ^203^	-
		BEN-28010	^204^	-
	CHEK2 (checkpoint kinase 2)	AZD7762	^205^	-
		N/A	^206^	-
	WEE1 (WEE1 G2 checkpoint kinase)	Adavosertib/MK-1775	^207-212^	NCT01849146, NCT02207010
		DEBIO-0123	(c)	NCT05765812
	CDC7 (cell division cycle 7)	PHA-767491	^213^ ^,^ ^214^	-
	PKMYT1 (protein kinase, membrane-associated tyrosine/threonine 1)	RP-6303	^215^ ^,^ ^216^	-
DNA replication, Fork protection and repair, DNA repair	RRM2 (ribonucleotide reductase regulatory subunit M2)	Triapine	^143^ ^,^ ^148^ ^,^ ^217^	NCT06410248
	NUDT1/MTH1 (nudix hydrolase 1/ MutT Homolog 1)	TH588	^218^	-
	RPA1 (replication protein A1)	HAMNO	^219^	-
	POLQ (DNA polymerase theta)	ART558	^220^	-
		Novobiocin, ART558	^150^	-
	PARP1/2 (poly(ADP-ribose) polymerase 1/2)	Pamiparib	^221^	NCT04614909, NCT03150862
		Talazoparib	^222^ ^,^ ^223^	NCT04740190
		Niraparib	^224^	NCT04715620, NCT01294735, NCT05076513, NCT04221503, NCT06258018, NCT06388733 (Gliofocus)
		Olaparib	^223^ ^,^ ^225^ ^,^ ^226^	NCT05463848, PARDIGM/ISRCTN52658296, PARADIGM-2/ ISRCTN51253312, NCT05432518, NCT02974621, NCT01390571/OPARATIC, NCT03212742
		Veliparib	^223^ ^,^ ^227^ ^,^ ^228^	NCT01026493, NCT02152982, NCT00770471, VERTU ACTRN12615000407594
		Rucaparib	^223^ ^,^ ^229^	NCT02711137: terminated due to safety issues
	PCNA (proliferating cell nuclear antigen)	ATX-100	^230^	-
	PARG (poly(ADP-ribose) glycohydrolase)	PDD00017273	^231^	-
	RAD51 (RAD51 recombinase)	RI-1	^232^	-
		B02	^233^	-
	RAD52 (RAD52 DNA repair protein)	L-OH-DOPA	^65^ ^,^ ^220^	-
	MRE11 (MRE11 double strand break repair nuclease)	Mirin	^234^	-
	REV1 (REV1 DNA directed polymerase)	JH-RE6	^235^	-
	FEN1 (flap structure-specific endonuclease 1)	FEN1-in-4	^236^	-
	TOP1 (DNA topoisomerase I)	Irinotecan	^237-241^	NCT00671801: Terminated because of high toxicity
		Topotecan	^242^	-
	TOP2A (DNA topoisomerase II alpha)	Etoposide	^243-245^	-
	USP1 (ubiquitin specific peptidase 1)	Pimozide	^246^ ^,^ ^247^	-
	TDP1 (tyrosyl-DNA phosphodiesterase 1)	N/A	^248^	-

Additional references: (a) https://doi.org/10.1093/neuonc/noad179.0307; (b) https://doi.org/10.1200/JCO.2025.43.16_suppl.TPS2100; (c) https://www.debiopharm.com/wp-content/uploads/2024/01/Debio-0123_poster-DDR-summit-2024_final-1.pdf.

Despite the initial disappointment provoked by the PARPi veliparib, which induced severe systemic and hematologic toxicity without significant survival improvement in GBM patients, several PARPi are now in clinical trials. The OPARATIC trial (NCT01390571) demonstrated that olaparib reaches therapeutic levels within the tumor area and is well tolerated in combination with TMZ, setting the stage for subsequent studies.^249^ Among these, the PARADIGM trial (ISRCTN52658296) identified olaparib as a potent radiosensitizer,^250^ while the ongoing PARADIGM-2 trial (ISRCTN51253312) is currently evaluating its efficacy in combination with TMZ and IR. Despite its good pharmacokinetic profile, olaparib’s adverse side effects prompted dose reductions and treatment adjustments. Subsequently, niraparib, which displayed a better pharmacokinetic profile than olaparib, has been considered a more promising agent in GBM.^251^^,^^252^ Gliofocus (NCT06388733), an ongoing study comparing the efficiency of TMZ and niraparib administered during the radiation period, then as monotherapy, in newly diagnosed GBM patients with unmethylated MGMT, represents a pivotal step in GBM therapy, having reached phase 3 clinical trial.

Other DDRi in clinical trials include inhibitors of ATM and DNA-PK. The 2 ATMi currently under evaluation exhibit favorable pharmacokinetic profiles and radiosensitizing potential. Following studies demonstrating significant radiosensitization in *TP53*-mutant GBM cell lines, a clinical trial of the highly selective DNA-PKi peposertib is now ongoing, with preliminary results suggesting a favorable safety profile and potent radiosensitizing effects in tumors with DDR mutations.^198^^,^^253^ Likewise, Wee1 inhibition sensitized *TP53*-mutant GBM cells to DNA-damaging agents,^254^ and the Wee1i debio-0123, which exhibited favorable brain to plasma ratio with no adverse systemic toxicities, is currently being evaluated in combination with RT + TMZ in newly diagnosed GBM, as well as in combination with TMZ in recurrent GBM (https://www.debiopharm.com/wp-content/uploads/2023/11/SNO-2023-poster.pdf).

Although the number of targets being investigated pre-clinically is quite extensive (Table 1) and several DDRi (eg, RI-1 for RAD51 or HAMNO for RPA) show great cytotoxicity in vitro,^219^^,^^232^ blood-brain barrier (BBB) penetration remains a constant hurdle. Notably, while trials may include newly diagnosed GBM, recurrent GBM, or both, they often limit genetic analysis to MGMT promoter methylation status. Under the prism of synthetic lethality, designing future clinical studies focusing on specific mutational landscapes (eg, HR deficiency, *TP53* mutations) could prove crucial for the development of successful clinical trials and a step forward for personalized medicine in GBM. Relevant in this context, pre-clinical studies indicate that ATM inhibition induces conditional lethality in *TP53*-mutant GBM, by targeting DSB repair in a G2/M checkpoint deficient background, thus specifically sensitizing these tumors—but not *TP53*-WT tumors—to TMZ and IR.^150^^,^^255^ Importantly, although a large spectrum of mutations, gene fusions and copy number variations affect the expression of oncogenes and tumor suppressors in GBM,^120^ the vulnerabilities and opportunities for synthetic lethality exposed by these alterations remain largely unexplored.^256-258^

### Overcoming Mechanisms of Resistance to DDR Inhibitors

Despite the success of PARP inhibitors in the clinic, most patients will develop resistance,^182^ as acquired resistance is a common feature of DDRi. Several resistance mechanisms (reviewed in Refs^173^^,^^182^^,^^259^^,^^260^) have been described, including (i) drug-target alterations, (ii) the bypassing of cell cycle checkpoint regulation, (iii) the activation of alternative DDR scenarios to restore DNA repair capacity, stabilize replication forks or tolerate lesions, (iv) the activation of mechanisms to evade programmed cell death, and (v) the activation of metabolic pathways altering cell fitness.^261^ Current strategies to overcome resistance involve combining multiple DDRi, as well as combining DDRi with immune-checkpoint inhibition or targeted therapies.^262-264^ Noteworthy, the evidence suggests that targeting vulnerabilities persisting in resistant tumors or resulting from the development of DDRi resistance may contribute to the eradication of resistant cells.^265^

### Tumor Treating Fields and the RSR

Recent years have seen the development of a noninvasive GBM therapy modality named Tumor Treating Fields (TTFields) that delivers low-intensity, intermediate frequency alternating electric fields to the tumor.^266^^,^^267^ TTFields alter cell cycle progression and disrupt mitosis, triggering CIN and cell death.^268^^,^^269^ Moreover, combining TTFields with RS-inducing drugs/DDRi increased chemotherapeutic efficacy, suggesting that TTFields also target RS.^270-272^ In line with this notion, TTFields elicited DSBs and R-loop-associated RS in non-small cell lung cancer (NSCLC) cell lines,^272^ as well as undermining the RSR in NSCLC and malignant pleural mesothelioma cell lines through the downregulation of the Fanconi-Anemia-BRCA pathway and DNA replication genes.^272^^,^^273^ In glioma, combining TTFields with IR or TMZ resulted in additive or synergistic effects, while TMZ-resistant cells were responsive to TTFields application.^269^ Mechanistically, TTFields were recently found to elicit PARP-mediated DNA repair and the activation of ATR signaling in glioma cells.^274^ Consequently, combining inhibition of PARP1 or ATR increased DNA damage and potentiated the cytotoxicity of TTFields alone or in combination with IR.^274^ As TTFields enhance BBB permeability,^275^ the authors have also proposed that TTFields could improve the effective dose of DDRi such as PARPi and ATRi at the tumor site.^274^

## Targeting the Replication Stress and DNA Damage Responses to Promote Immunotherapy in Glioblastoma

Although the central nervous system (CNS) displays a distinct immunological environment, it is still capable of mounting efficient immune responses. The CNS is populated mostly by antigen-presenting cells called brain-resident macrophages, also known as microglia (reviewed in Ref^276^). Upon CNS damage, such as neuroinflammation or brain malignancies, resident microglia expansion is observed, alongside the recruitment of bone marrow-derived monocytes that differentiate into phenotypically distinct cells from endogenous microglia. Furthermore, the presence of brain tumors can disrupt the BBB, leading to an increased infiltration of immune cells in the tumor area. Patients with high-grade glioma that undergo standard-of-care treatment experience systemic immunosuppression, such as CD4+ lymphopenia, due in part to the administration of a synthetic glucocorticoid, dexamethasone, which decreases the overall immune response significantly. Besides systemic immunosuppression, which leads to a higher risk of infections and constrains immunotherapy approaches,^277^^,^^278^ GBM patients also undergo tumor-driven alterations to the brain immune system through immunosuppressive cytokines and chemokines, and the modulation of cell surface receptors and immune cell subsets.^277^^,^^278^

GBM has long been considered an immunologically cold tumor due to low T cell infiltration and overexpression of colony-stimulating factor-1 (CSF-1), thus recruiting and modifying immune cells to foster a pro-tumorigenic phenotype characterized by an immunosuppressive tumor microenvironment (TME) (reviewed in Refs^276-278^). Noteworthy, the GBM TME is characterized by the accumulation of tumor-associated microglia/macrophages (TAMs). The systemic and localized immunosuppression assumed by GBM, compounded by a BBB refractory to large molecules, has limited the efficiency of immunotherapeutic approaches, such as immune checkpoint blockade (ICB), in contrast to several “hot” tumor types with a responsive immune niche where immunotherapy has proved a game changer.^279^^,^^280^ While neoadjuvant immunotherapy administered as a first-line treatment before surgical resection has recently been considered in GBM,^281^ most current efforts focus on combination strategies to stimulate immune cells into a reactive state needed to overcome the cold nature of GBM’s TME.^282^^,^^283^ Targeting the DDR to promote immunotherapy has evolved as a promising strategy in this context. Central to this concept is the recognition of self-nucleic acids by the innate immune system.

### Self-Nucleic Acids in Innate Immunity

The innate immune response is triggered by the recognition of foreign or “non-self” agents by pattern recognition receptors (PRRs), and proceeds through signaling cascades that activate key transcription factors (eg, NF-κB and IRF3), leading to the production of pro-inflammatory cytokines, chemokines, type I interferons (IFN-I), and interferon-stimulated genes (ISGs) that attract immune cells and induce cell death.^284^ Other mechanisms can trigger innate immunity, such as the assembly of the inflammasome complex triggered by the engagement of the dsDNA sensor AIM2, which elicits the maturation and secretion of the pro-inflammatory cytokines IL-18 and IL-1β, and the induction of Caspase-1-mediated cell pyroptosis.^285^

Among the molecules recognized by PRRs are pathogen-associated molecular patterns (PAMPs) such as nucleic acids, including dsRNA and dsDNA with hypomethylated CpG motifs.^284^^,^^286^ Additionally, the innate immune system is able to detect damage-associated molecular patterns (DAMPs) stemming from cellular stress, injury, or death, including a large panel of immunostimulatory self-nucleic acids generated following DNA damage or as intermediates or by-products of DNA replication, repair, and recombination, and which have escaped tolerance mechanisms.^287-294^ The evidence indicates that self-nucleic acids can trigger an innate immune response by various mechanisms.

### Induction of Innate Immune Response by Accumulation of Self-Nucleic Acids in the Cytoplasm and Activation of Cytosolic Sensors

**Figure 5.**
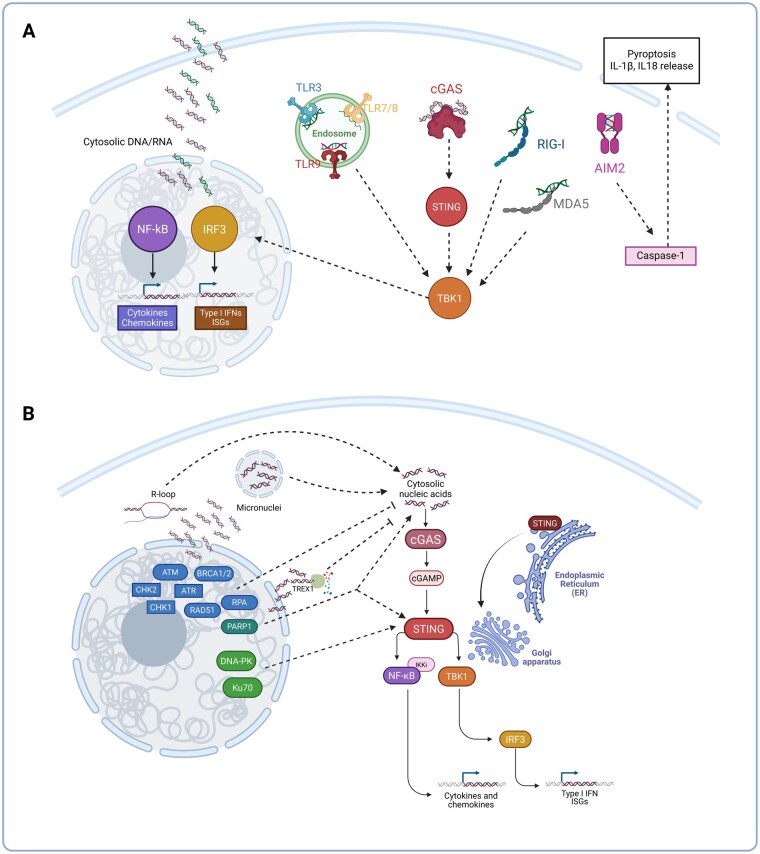
Innate immune sensors and their interplay with the DDR/RSR. (A) DNA/RNA recognition by innate immune sensors. Both RNA sensors such as MDA5, RIG-I, TLR3, TLR7/8 and DNA sensors like cGAS, AIM2, and TLR9 are able to detect the accumulation of self-nucleic acids in the cytoplasm, which in turn leads to the stimulation of a STING-TBK1 response that induces translocation of IRF3 and NF-κB to the nucleus and release of type-I IFN, ISGs (Interferon stimulated genes), cytokines, and chemokines. Besides, the assembly of the inflammasome complex driven by AIM2, followed by the maturation and secretion of the pro-inflammatory cytokines IL-18 and IL-1β, results in the induction of cell pyroptosis via caspase-1. (B) The cGAS-STING pathway and its interplay with the DDR/RSR. The cGAS protein catalyzes the production of cyclic guanosine monophosphate-adenosine monophosphate (cGAMP), that in turn activates the STING protein present in the endoplasmic reticulum (ER), eliciting its oligomerization and translocation to the Golgi apparatus, where STING activates its downstream target TBK1. In turn, TBK1 phosphorylates IRF3, leading to IRF3 translocation to the nucleus where it acts as a transcriptional activator of type I IFN genes. The NF-κB pathway can be directly activated in a TBK1-dependent manner or indirectly via STING-mediated activation of inhibitor of nuclear factor kappa B kinase (IKK), in a TBK1-independent manner. A variety of RSR/DDR factors have been described to directly or indirectly induce the production of type-I IFN, ISGs (Interferon-stimulated genes), cytokines and chemokines, either by direct STING activation (green) or when their loss of function (blue) results in the accumulation of nucleic acids that activate a cGAS-STING immune response, respectively. Besides, micronuclei accumulation due to chromosome instability has also been reported to stimulate the cGAS-STING pathway. Identified in squares are the DDR factors whose inhibition has been reported to lead to an immune response in GBM.

Self-nucleic acids that accumulate in the cytoplasm can be recognized by a variety of RNA sensors (eg, MDA5, RIG-I, TLR3, TLR7, TLR8) and DNA sensors (eg, ZBP1/DAI, IFI16, DDX41, AIM2, TLR9).^286^^,^^295^ In addition, the cGAS-STING pathway (cyclic GMP-AMP synthase (cGAS)-stimulator of interferon genes (STING)), where cGAS acts as a sensor of cytosolic dsDNA, plays a major role in the recognition of pathogenic and self-DNA (Figure 5A).^286^^,^^289^^,^^296^

Investigation of the mechanisms whereby the cGAS-STING pathway connects DNA damage with immune and inflammatory responses has revealed its complex interplay with RSR/DDR components (Figure 5B). In Table 2, we provide a list of the DDR factors whose activity prevents the accumulation of nucleic acids and/or the activation of an immune response. Notably, the cytoplasm hosts the DNA exonuclease TREX1, which acts as a sentinel to degrade self and foreign DNA, thus providing an additional layer of protection from excessive cGAS-STING-mediated cytosolic DNA sensing.^297^ Moreover, direct partners of the cGAS-STING pathway in innate immunity, such as the nuclease ENDOD1, have been found to also exert a DNA repair function in the nucleus, demonstrating the strong interplay between these processes.^298^

**Table 2.** List of RSR/DDR factors, their respective pathways and their impact on immune responses

DDR factor	Pathway	Impact on immunity	References
DNA-PK	NHEJ, DSBR, DDR apical kinase	Is detected in the cytoplasm where it was shown to bind to cytosolic dsDNA, activating a STING-TBK1-IRF3-dependent IFN-I response. Reported to activate a STING-independent DNA-sensing pathway in human cells by yet-unknown mechanisms. Human fibroblasts and mouse models lacking DNA-PKcs display an attenuated cytokine response to both DNA and DNA viruses.	^3^ ^,^ ^299-303^ ^304^ ^,^ ^305^
Ku70	NHEJ, DSBR	Reported to translocate into the cytoplasm, where it is able to sense transfected or viral DNA to trigger an anti-viral STING-dependent response mediated by IRF1/IRF7 and the release of type III IFN - IFN-λ1 signaling.	^301^ ^,^ ^302^ ^306-308^
RAD51	HR, DSBR, Fork protection	Inhibition of RAD51 leads to replication stress, loss of protection from MRE11 exonuclease and failure to repair replication forks. Moreover, it leads to the accumulation of DNA fragments in the cytosol, eliciting an innate immune response mediated by cGAS-STING.	^87^ ^,^ ^289^ ^,^ ^309^
BRCA2	HR, FA, DSBR	BRCA2 inactivation leads to cytosolic nucleic acid accumulation and cGAS-STING-mediated interferon response. Cancer cells defective in BRCA2 display enhanced TNFa sensitivity.	^289^ ^,^ ^310^ ^,^ ^311^
BRCA1	HR, FA, DSBR, transcription	BRCA1-mutant breast cancers are characterized by lymphocytic infiltration. BRCA1 loss leads to the accumulation of cytosolic DNA that leads to the activation of a cGAS-STING response.	^89^ ^,^ ^90^ ^,^ ^311^
ATM	Apical DDR/RSR kinase	Clinically, mutations in ATM cause ataxia telangiectasia (A-T) syndrome, a cancer-prone neurological disease associated with persistent genome instability that leads to chronic inflammation and autoimmune symptoms. Accumulation of cytoplasmic DNA and stimulation of a cGAS-STING inflammatory response is observed in fibroblasts from A-T patients, ATM-deficient microglia, as well as upon ATM inhibition in a variety of cancers. Animal experiments showed that ATM-deficient tumors displayed increased PD-L1 expression, higher infiltration of T-cells and better survival.	^306^ ^,^ ^312^ ^284^ ^,^ ^313-319^
ATR	Apical DDR/RSR kinase	ATR was reported to play a role in IFN signaling. Inhibition of ATR potentiated an ionizing radiation-induced innate immune response in epithelial cell lines. ATRi (AZD6738) combined with conformal radiotherapy (RT) induced a durable CD8+ T cell–dependent antitumor response, in murine cancer models. ATR inhibition led to cytoplasmic DNA and RNA accumulation, leading to activation of the cGAS/STING-dependent and/or MAVS-dependent RNA sensing pathways. ATR inhibition led to an enhanced IFN signaling in GBM cells after X-ray, proton and carbon ion irradiation.	^306^ ^,^ ^312^ ^4^ ^,^ ^43^ ^,^ ^313-322^
PARP	SSBR, DSBR, Fork protection, transcriptional co-activator	PARP inhibition promoted accumulation of cytosolic DNA fragments and the activation of the cGAS-STING pathway and stimulated production of type I interferons to induce tumor infiltrating lymphocytes (TILs) and antitumor immunity, in mouse models of ovarian and colon cancer. PARPi can be combined with immune checkpoint blockade therapies, to increase their efficacy. As a transcriptional co-activator, PARP1 induces the translocation of NF-κB into the nucleus and stimulates the NF-κB-dependent expression of pro-inflammatory factors such as TNFα, IL-6 or iNOS.	^323-326^
RPA	DNA repair, Fork protection	Loss of RPA, alongside RAD51, leads to the increased accumulation of short ssDNA in the cytosol. A cGAS-dependent type I IFN response is activated due to this accumulation. Deletion of RPA1 also activates a ZBP1-RIPK3 signaling that leads to necroptotic cell death of proliferative T cells, which results in lymphopenia and a strong pro-inflammatory innate immune response.	^327^ ^,^ ^328^
CHK1	DSBR	ARID1A-deficient CRC cells exposed to radiation therapy and treated with a CHK1i (CCT244747) show accumulation of ssDNA in the cytosol and activation of the cGAS-STING-type I IFN pathway. Pharmacological inhibition of Chek1 (Prexasertib) sensitized glioma models to immune checkpoint blockade with PD-1.	^205^ ^,^ ^329^
CHK2	DSBR	Both depletion (KO) and inhibition (AZD7762) of CHEK2 sensitized glioma tumor cells to CD8+ T-cell mediated killing and to immune checkpoint blockade with PD-1, by enhancing antigen presentation and increasing a type I-IFN response.	^205^

### Anti-Cancer Immune Responses Triggered by DNA Damage Signaling

While strategies to elicit immunogenic cell death (ie, stimulation of the immune system by DAMPS exposed by dead cells) have evolved as a major anti-cancer approach,^330^ recent studies have pointed to signaling pathways activated by DNA-damaging chemotherapeutics in live, DNA-damaged tumor cells as drivers of an anti-cancer immune response, thus suggesting other approaches to stimulate the immune system. Specifically, tumor cells surviving chemotherapeutic DNA-damaging agents were found to activate the apical DDR kinases ATR, ATM, and DNA-PK, as well as NF-κB-, MAPK-, and RIPK1-mediated signaling, eliciting the activation of immune T-cells.^331^ How these signaling pathways increase tumor cell immunity remains to be elucidated.

### Induction of an Innate Immune Response by Accumulation of Micronuclei

The activation of the cGAS-STING pathway by cytosolic DNA has also been proposed to occur following rupture of MN and the release of their DNA cargo in cells exhibiting RS and chromosome instability.^289^ Rupture of the MN envelope allows the release of DNA in the cytoplasm,^332^ and initial studies of MN in cells exposed to DNA damage led to the conclusion that MN constituted a repository of immunostimulatory DNA recruiting cGAS and eliciting cGAS-STING-mediated signaling.^333^^,^^334^ However, the activation of cGAS after recognition of MN has recently been questioned in a study showing that, while cGAS was indeed recruited to MN in response to IR, RS and missegregated chromosomes, it failed to activate the cGAS-STING pathway in several immortalized and primary non-cancer and cancer cell lines.^335^ These results have been supported by recent studies showing that MN induced by a panel of antimitotic drugs or sister-chromatid fusions also failed to induce a cGAS-STING immune response.^336^^,^^337^

### Induction of an Innate Immune Response by R-loop Formation

Defects in the helicase Senataxin involved in R-loop removal elicited cGAS-mediated stimulation of interferon genes.^293^ Besides, the aberrant processing of R-loops led to the release of cytoplasmic RNA-DNA hybrids that were recognized by both cGAS and TLR3, driving an IRF3-mediated immune response.^292^ The mechanisms orchestrating the processing of R-loops and their processing and their translocation from the nucleus are still obscure.^287^

### Targeting cGAS-STING and Other PRRs in Cancer and GBM

The link between innate immune response and RSR/DDR has implications for cancer therapy. Thus, DDRi are undergoing evaluation as monotherapies or in combination with immune checkpoint inhibitors to overcome therapeutic resistance and promote effective antitumor immunity (reviewed in Refs^264^^,^^338^). Likewise, exploiting defective PRRs has been considered in strategies using DNA-damaging agents. Thus, low expression of cGAS and STING has been observed in several cancer types, including ALT-positive tumors and CNS tumors, leading to the notion that immune responses expected to arise from DNA damage in cancer cells resulting, for example, from radio-chemotherapies, may be suppressed upon cGAS/STING loss.^339-341^ Non-neoplastic brain cells have a hyper-methylated STING promoter, suggesting that the epigenetic silencing of STING is characteristic of CNS cells under normal physiological conditions.^339^^,^^340^ Such silencing is also observed in GBM, where it may contribute to the established immunosuppressive environment associated with GBM. Treatment with the DNA demethylation agent decitabine (an inhibitor of DNA methyltransferases) rescues STING expression in GBM cell lines; however, the potential of this re-expression in antitumor immune response or sensitization of tumor to immunotherapy has yet to be explored.^339^ Nonetheless, STING is expressed in immune cells present in the TME of GBM, such as the myeloid cells, which makes antitumor approaches based on the induction of the cGAS-STING pathway interesting to explore.^340^ One such approach is the use of STING agonists that increase infiltration of innate immune cells and immune stimulation in the GBM TME. Even though these agonists have not yet been tested in human clinical trials, promising results have been seen in animal models.^342-345^

Silencing of STING resulting in defective cytosolic DNA sensing is also observed in ALT-positive cancer cells.^345^ Among the defining features of ALT are the accumulation of extrachromosomal telomere repeats (ECTRs), as well as the accumulation of TERRA lncRNAs.^26^ The induction of ECTRs has been shown to elicit a cGAS-STING response, triggering an innate immune response in normal human fibroblasts, suggesting that the loss of cGAS-STING signaling is required to evade the adverse effect of ECTR accumulation, which could be exploited therapeutically.^345^ Likewise, recent evidence indicates that the imposition of transcriptional stress at telomeres in mouse embryonic fibroblasts can elicit the accumulation of R-loops and the cytosolic release of telomeric DNA fragments capable of triggering an innate immune response and senescence in neighboring cells.^346^ Finally, G4 binders that stabilize G-quadruplexes have been shown to function as cytostatic modulators of innate immune genes in cancer cells,^347^ and the development of G4 ligands capable of triggering cytoplasmic DNA/RNA release to stimulate antitumor immunity is under consideration.^348^ The implementation of strategies targeting telomeric R-loop and G-quadruplex in GBM awaits further studies.

### DDR Alterations and the Immune Landscape in Glioma

Recent publications using DDR scoring methods have provided evidence that DDR evaluation may help optimize immunotherapy in GBM. Analysis of the alterations affecting 140 DDR genes, as well as tumor microenvironment signatures in glioma samples from TCGA, revealed correlations between DDR alterations, immune phenotypes (including infiltrating immune cell types and immunosuppression) and cytokine-associated pathways.^349^ Noteworthy, established TMZ-resistant GBM cells (selected upon extended exposure to TMZ in vitro) displayed higher DDR scores than their parental cell lines. Moreover, the expression of DDR-related cytokines (C5, SAA1, TNFSF4, IL6, MDK, VEGFA) was higher in the TMZ-resistant cell lines, as well as in their related xenografts, than in parental cell lines, and the evidence suggested that DDR changes were associated with macrophage polarization toward a tumor-supportive phenotype.^349^ The increased DDR gene expression seen in established TMZ-resistant GBM cell lines was confirmed recently.^350^ In addition, this study developed a novel DDR score—derived from 9 differentially expressed genes between TMZ-sensitive/resistant cells—which acted as a prognostic indicator across TCGA and CGGA datasets, distinguishing distinct tumor microenvironment features and tumor immunogenicity profiles.^350^ Moreover, genetic screens in GBM have also revealed that the depletion of several DDR factors, such as LIG4, MRE11A, and BRCA2, was associated with the expression of surface receptors and soluble factors capable of increasing both infiltration of NK cells and their activation, leading to secretion of IFN-γ and increased cytotoxicity.^351^ Finally, in vivo screening of human glioma kinases identified CHK2 as the most important kinase fostering evasion of GBM cells from CD8 T cell recognition.^205^ Noteworthy, CHK2 depletion/inhibition in combination with PD-1 blockade led to increased antigen presentation and PD-L1 expression, as well as the activation of the STING pathway in mouse gliomas. These observations add weight to the notion that DDR/RSR factors could be exploited in order to optimize the response of GBM to immunotherapies, by creating a T-cell abundant TME and therefore reversing its status of “cold” tumor.^205^

## Perspectives

As this review underscores, the strategic reasons for targeting RS in GBM appear to be numerous, including (i) to enhance the efficacy of therapeutic approaches based on RS-inducing drugs and prevent treatment resistance, (ii) to exploit crucial features of GBM tumors and their niches, and (iii) to promote anti-cancer immunity. GBM is driven by alterations including genetic mutations and copy number variations that play crucial roles in the expression of oncogenes and tumor suppressors, including DDR genes. Comprehensive knowledge regarding the impact of these alterations on the RSR, as well as the spectrum of vulnerabilities that they expose for synthetic lethality, is still in early stages. In this regard, although glioma cell subpopulations have been shown to undergo chronic RS, further studies will be necessary to understand how RS affects the distinct glioma cell states and to what extent the RSR is impacted by the glioma cell state and/or localization within the brain and the various tumor niches. As RS responses are shaped by molecular alterations and gene expression programs, it is likely that knowledge of the molecular landscape of RSR in GBM will reveal differences and redundancies in scenarios among GBM cells. We anticipate that such knowledge will have a profound direct impact on instructing precision medicine in GBM, for instance by predicting sensitivity/resistance to DDR inhibitors.

The barrier imposed by the BBB has restricted the use of several RS-inducing drugs and is a major hurdle in the use of DDRi.^352^^,^^353^ Thus, it remains of utmost importance to develop new methods of delivering promising drugs into the brain. Several technologies are being developed, including (i) TTFields, (ii) nanoparticles used as delivery systems, (iii) intranasal administration, (iv) ligand-conjugation for brain targeting, (v) optimized drug design to enhance lipophilicity, and (vi) temporal disruption via light or ultrasounds.^352^^,^^354^ In this regard, encouraging results were reported when TMZ^355^^,^^356^ or cisplatin^357^ were combined with microbubble-enhanced focused ultrasound (MB-FUS), a technology that is currently in clinical trials in patients with recurrent GBM, with paclitaxel and carboplatin (https://clinicaltrials.gov/study/NCT04528680). Similarly, technologies encapsulating RS-inducing drugs (eg, doxorubicin),^358^ mitotic inhibitor (eg, paclitaxel),^359^ or DDRi (Topo1i)^360^^,^^361^ in nanoparticles conjugated/coated with tumor cell-targeting molecules such as ferritin (targeting transferrin) or cyclo(Arg-Gly-Asp-D-Phe-Cys) (targeting *α*_v_*β*_3_-integrin), showed good BBB penetration and specific delivery to glioma cells, resulting in tumor volume reduction and increased survival rates in various GBM models. Taking into consideration the reported cytotoxicity of several DDRi, it is important to mention that the increase of BBB penetration will allow for the use of lower levels of drugs.^362^ Furthermore, developing drug analogues that maintain optimal therapeutic efficacy while demonstrating reduced systemic toxicity will be essential to improving clinical outcomes.

Finally, new interest in the interplay between DDR and the immune response is emerging. The use of DDRi for the recruitment of immune cells into the TME and the stimulation of immune responses and checkpoint blockade marker expression is showing promise in GBM, instilling hopes that exploiting RS and harnessing the RSR/DDR will promote strategies fostering antitumor immunity.
